# New-Onset Convulsive Status Epilepticus Temporally Associated With Accidental Inhalation of Billygoat Weed (Ageratum conyzoides) Smoke: A Case Report

**DOI:** 10.7759/cureus.113060

**Published:** 2026-07-20

**Authors:** Prajwal Gowda, Ranvijay Singh, Namit Sairam, Ashutosh Lakade

**Affiliations:** 1 Internal Medicine, All India Institute of Medical Sciences, Bilaspur, Bilaspur, IND

**Keywords:** ageratum conyzoides, billygoat weed, neurotoxicity, refractory status epilepticus, seizure, smoke inhalation, status epilepticus (se)

## Abstract

Status epilepticus is most commonly associated with structural, metabolic, infectious, autoimmune, or toxic etiologies. Plant-related toxic exposures are uncommon causes of new-onset status epilepticus and remain poorly characterized. Ageratum conyzoides (billygoat weed) is an invasive plant widely distributed in tropical and subtropical regions and is frequently used in traditional medicine. We report the case of a 76-year-old male with no prior history of epilepsy, cerebrovascular disease, dementia, head injury, alcohol use, or substance abuse who developed generalized convulsive status epilepticus approximately five minutes after the accidental inhalation of smoke generated from burning Ageratum conyzoides. He presented to the emergency department with ongoing seizures requiring treatment with intravenous benzodiazepines, endotracheal intubation, and ICU admission.

Extensive investigations, including cerebrospinal fluid analysis, metabolic profile, thyroid function testing, renal and liver function tests, autoimmune workup, urine toxicology, blood ketone levels, and carbon monoxide levels, were unremarkable. Contrast-enhanced MRI of the brain demonstrated diffuse cerebral atrophy with Fazekas grade 2 white matter changes but without evidence of an acute structural lesion to account for the seizures. Electroencephalography (EEG) performed following stabilization was normal. The patient's condition improved clinically; he was successfully extubated and discharged after a three-day ICU stay. He remained neurologically intact and seizure-free at the three-month follow-up. This report highlights a possible association between Ageratum conyzoides smoke inhalation and new-onset status epilepticus and underscores the importance of considering environmental toxin exposure in patients presenting with unexplained seizures.

## Introduction

Status epilepticus is a life-threatening neurological emergency characterized by prolonged seizure activity and is associated with substantial morbidity and mortality [1]. Acute symptomatic status epilepticus may result from metabolic abnormalities, infections, cerebrovascular disease, autoimmune disorders, structural brain lesions, and toxic exposures [1,2]. Identification of the underlying etiology remains essential for appropriate management and the prevention of recurrence.

Status epilepticus is broadly classified into convulsive and non-convulsive forms depending on whether prominent motor activity is present. Convulsive status epilepticus involves sustained or repeated generalized tonic-clonic seizure activity with impaired consciousness. Non-convulsive status epilepticus, by contrast, lacks overt motor signs and instead presents as altered mental status or subtle behavioral changes. Because clinical features alone are often insufficient, electroencephalography (EEG) confirmation is generally required for diagnosis.

Clinical decision-making in status epilepticus is now guided by two operational time points introduced in the 2015 International League Against Epilepsy (ILAE) framework: T1, the point at which a seizure is unlikely to stop spontaneously and treatment should begin, and T2, the point beyond which the risk of lasting neuronal injury rises and more aggressive treatment is warranted [3,4]. For generalized convulsive status epilepticus specifically, T1 is set at five minutes and T2 at 30 minutes. Other seizure subtypes, such as focal status epilepticus with impaired awareness, have different thresholds reflecting their distinct clinical course.

Ageratum conyzoides, commonly known as billygoat weed, is a widely distributed invasive herb belonging to the Asteraceae family [5]. The plant has been extensively used in traditional medicine for wound healing, gastrointestinal disorders, and inflammatory conditions [5,6]. Phytochemical studies have identified several biologically active compounds, including pyrrolizidine alkaloids, flavonoids, coumarins, and essential oils [5-7]. While hepatotoxic and genotoxic effects of some constituents have been described, evidence of acute neurological toxicity in humans remains limited [7].

Although Ageratum conyzoides has been investigated for its medicinal properties and toxic effects, reports describing acute neurological complications following the inhalation of smoke from this plant are lacking. To the best of our knowledge, convulsive status epilepticus occurring shortly after accidental exposure to Ageratum conyzoides smoke has not been described previously in the medical literature. We present this case to highlight a possible association and to encourage awareness of an uncommon environmental exposure that may warrant further investigation.

## Case presentation

A 76-year-old male was brought to the emergency department with ongoing generalized tonic-clonic seizures. On presentation, the patient was hemodynamically stable with a blood pressure of 130/80 mmHg, a pulse rate of 102 beats/min, a respiratory rate of 18 breaths/min, an oxygen saturation of 96% on room air, and a random blood glucose level of 148 mg/dL. Arterial blood gas analysis revealed mild lactic metabolic acidosis characterized by a decreased pH, elevated serum lactate, and mildly reduced bicarbonate levels, while PaO₂, PaCO₂, and oxygen saturation remained within normal limits.

According to family members, the patient had accidentally inhaled smoke generated from burning Ageratum conyzoides approximately five minutes before the onset of symptoms. There was no preceding history of fever, headache, recent infection, head trauma, alcohol consumption, recreational drug use, medication exposure, cerebrovascular disease, dementia, or epilepsy. The exposure occurred outdoors while Ageratum conyzoides was being burned for vegetation clearance. According to the attendants, the patient was in close proximity to the burning plant and was exposed to the smoke for a short period before developing symptoms. There was no history of intentional inhalation, occupational exposure to industrial chemicals or pesticides, or recent exposure to other known neurotoxic agents. Although the exact duration and intensity of smoke exposure could not be quantified, generalized convulsive seizures developed within approximately five minutes of exposure.

On arrival, the patient remained in convulsive status epilepticus. Initial stabilization was undertaken, and treatment was initiated with intravenous lorazepam 4 mg (0.1 mg/kg), which was repeated once for persistent seizure activity, in accordance with first-line benzodiazepine dosing recommendations for status epilepticus. As seizures continued despite benzodiazepine therapy, second-line therapy was initiated with intravenous levetiracetam 60 mg/kg, followed by a 200 mg loading dose of intravenous lacosamide, consistent with American Epilepsy Society guideline-recommended second-line agents for established status epilepticus. Due to persistent seizure activity and the inability to maintain airway protection, endotracheal intubation was performed, and the patient was admitted to the ICU.

Laboratory investigations (Table 1) demonstrated normal serum sodium, calcium, magnesium, random blood glucose, thyroid function tests, renal function tests, liver function tests, serum ammonia, and blood ketone levels. Inflammatory markers, including erythrocyte sedimentation rate (ESR), C-reactive protein (CRP), ferritin, and lactate dehydrogenase (LDH), were within normal limits. Cerebrospinal fluid examination revealed no evidence of infection or inflammatory pathology. Antinuclear antibodies, ENA blot, autoimmune encephalitis panel, and extended viral panel were negative. Serum pseudocholinesterase levels and toxin screening for heavy metals were negative.

**Table 1:** Summary of laboratory investigations AST: aspartate aminotransferase; SGOT: serum glutamic-oxaloacetic transaminase; ALT: alanine aminotransferase; SGPT: serum glutamic-pyruvic transaminase; TSH: thyroid-stimulating hormone; ESR: erythrocyte sedimentation rate; CRP: C-reactive protein; LDH: lactate dehydrogenase; CSF: cerebrospinal fluid

Parameter	Patient value	Reference range
Hemoglobin	13.8 g/dL	13–17 g/dL
Total leukocyte count	7,800 /µL	4,000–11,000 /µL
Platelet count	2.3 × 10^5 ^/µL	1.5–4.5 × 10^5^ /µL
Serum sodium	138 mmol/L	135–145 mmol/L
Serum potassium	4.2 mmol/L	3.5–5.0 mmol/L
Serum calcium	9.1 mg/dL	8.5–10.5 mg/dL
Serum magnesium	1.9 mg/dL	1.7–2.2 mg/dL
Random blood glucose	148 mg/dL	70–140 mg/dL
Blood urea	18 mg/dL	7–20 mg/dL
Serum creatinine	1.0 mg/dL	0.6–1.3 mg/dL
AST (SGOT)	28 U/L	10–40 U/L
ALT (SGPT)	32 U/L	7–56 U/L
Serum ammonia	32 µg/dL	15–45 µg/dL
Serum lactate	3.2 mmol/L	0.5–2.2 mmol/L
Bicarbonate (HCO3-)	19 mmol/L	22–28 mmol/L
TSH	2.1 mIU/L	0.4–4.0 mIU/L
ESR	12 mm/hr	<20 mm/hr
CRP	2.1 mg/L	<5 mg/L
Ferritin	180 ng/mL	30–400 ng/mL
LDH	210 U/L	140–280 U/L
Blood ketones	0.3 mmol/L	<0.6 mmol/L
CSF cells	0 cells/mm³	0–5 cells/mm³
CSF protein	38 mg/dL	15–45 mg/dL
CSF glucose	72 mg/dL	50–80 mg/dL

**Figure 1:**
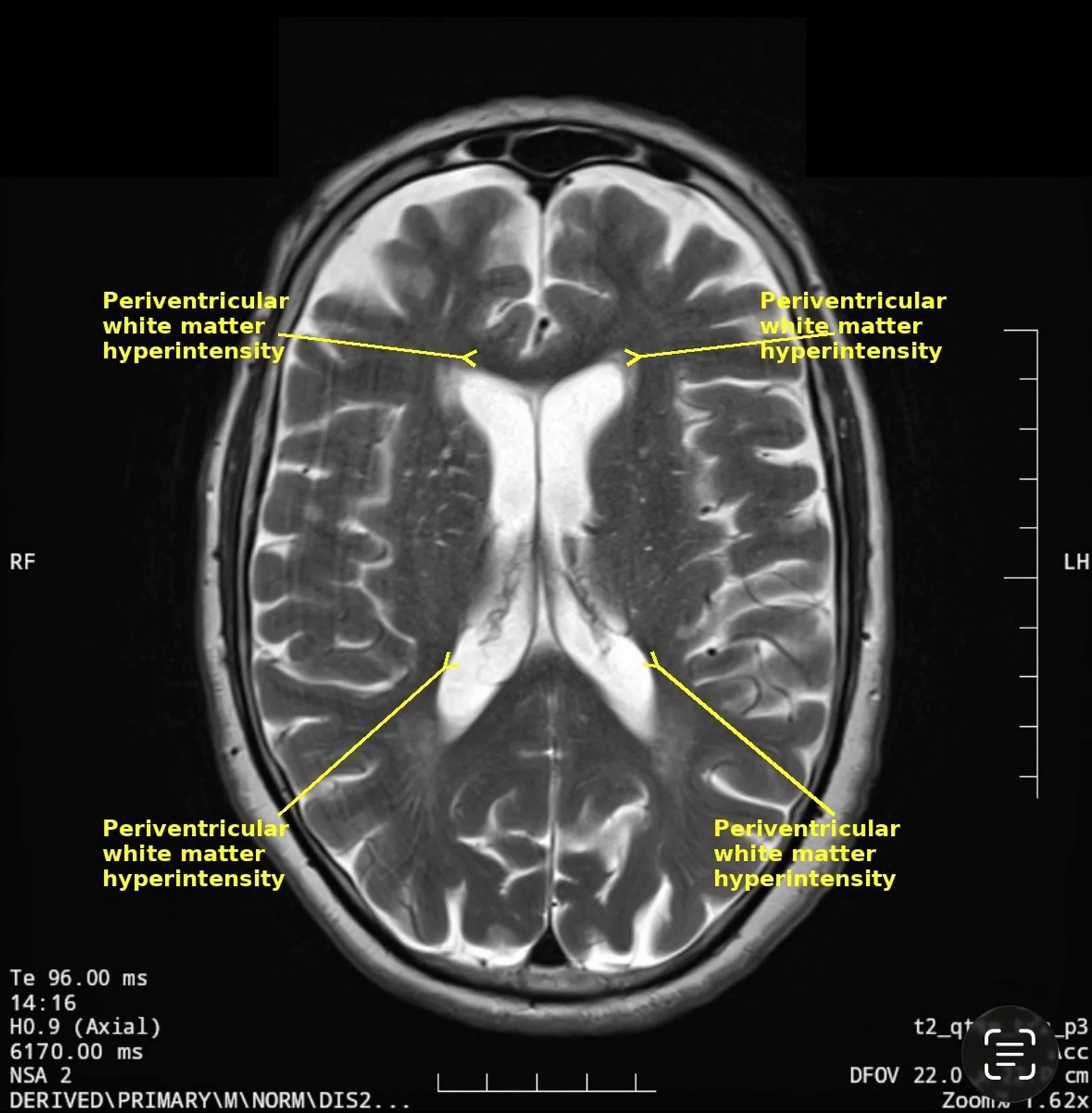
Axial T2-weighted MRI of the brain The image shows diffuse cerebral atrophy with periventricular white matter hyperintensities consistent with Fazekas grade 2 changes. No acute infarct, hemorrhage, or focal lesion is identified MRI: magnetic resonance imaging

Contrast-enhanced MRI of the brain demonstrated diffuse cerebral atrophy with Fazekas grade 2 white matter changes without evidence of acute ischemia, intracranial hemorrhage, encephalitis, or space-occupying lesions (Figure 1) [8]. A routine 40-minute EEG performed following clinical stabilization and seizure control showed no epileptiform abnormalities.

The patient remained in the ICU for three days. No further seizures were observed following the initiation of treatment. Neurological recovery was complete, and the patient was discharged in stable condition. He remained neurologically intact and seizure-free at the three-month follow-up.

## Discussion

**Figure 2:**
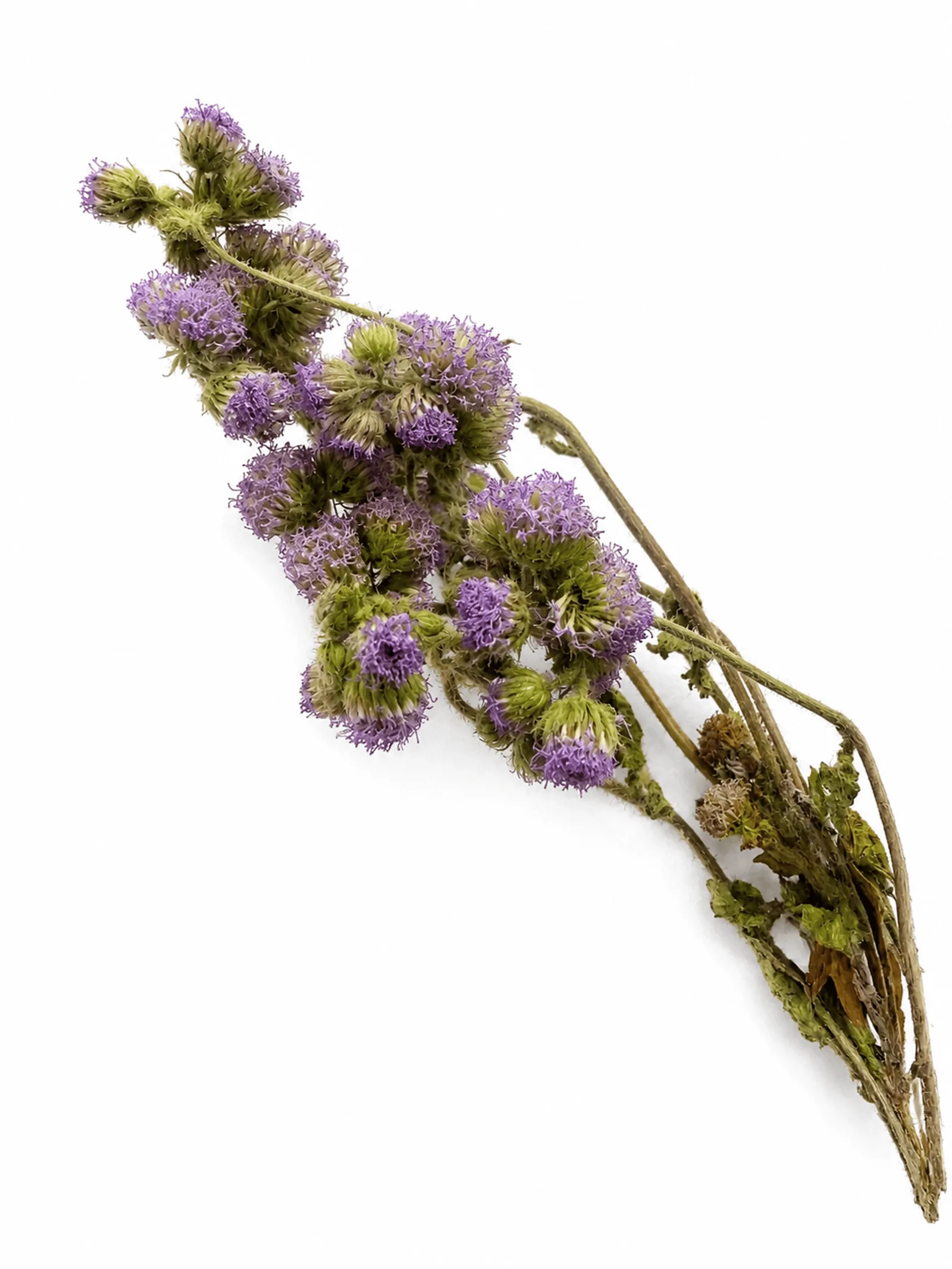
Ageratum conyzoides (billygoat weed) Ageratum conyzoides is an invasive herb belonging to the Asteraceae family, commonly found in tropical regions and used in traditional medicine Photograph by the authors

Toxic exposure is an important but often underrecognized cause of acute symptomatic seizures and status epilepticus [1,2]. Establishing causality can be challenging because diagnosis frequently relies on a temporal association and exclusion of alternative etiologies. Ageratum conyzoides (Figure 2) contains several bioactive compounds, including pyrrolizidine alkaloids, flavonoids, chromenes, and terpenoids [5-7]. Although the plant has been widely studied for its medicinal properties, reports describing acute neurological manifestations in humans are scarce [5,6]. Furthermore, the toxicological effects of combustion products generated from burning the plant remain poorly understood.

The present case is notable because seizure onset occurred within five minutes of smoke inhalation. Extensive investigations failed to identify an alternative explanation for status epilepticus. A systematic evaluation was undertaken to identify the underlying cause of convulsive status epilepticus. Metabolic etiologies, including abnormalities of blood glucose, serum electrolytes, calcium, magnesium, renal function, liver function, serum ammonia, and blood ketone levels, were excluded by laboratory investigations. Endocrine causes were considered unlikely given normal thyroid function tests. Infectious etiologies were excluded based on unremarkable cerebrospinal fluid analysis and a negative extended viral panel.

Autoimmune encephalitis was considered unlikely given the negative results of the autoimmune encephalitis panel, antinuclear antibody testing, and ENA blot analysis. Contrast-enhanced MRI revealed no evidence of acute ischemia, intracranial hemorrhage, encephalitis, or space-occupying lesions. Toxicological evaluation, including carbon monoxide assessment, heavy metal screening, and serum pseudocholinesterase levels, did not identify an alternative toxic etiology. In the absence of another identifiable cause, the close temporal relationship between accidental inhalation of Ageratum conyzoides smoke and seizure onset suggests a possible association; however, a causal relationship cannot be established based on a single case.

Although MRI demonstrated diffuse cerebral atrophy and Fazekas grade 2 white matter changes, these findings are relatively common in elderly individuals and were unlikely to account for the abrupt onset of convulsive status epilepticus. The exact mechanism underlying this presentation remains uncertain. Potential explanations include direct neurotoxicity from plant-derived compounds released during combustion, exposure to unidentified combustion products, or an idiosyncratic neurological response to inhalational exposure.

While the temporal association between Ageratum conyzoides smoke inhalation and the onset of status epilepticus is notable, a definitive causal relationship cannot be established from a single case report. This report has several limitations. Toxicological analysis of the inhaled smoke was not performed, and the precise exposure dose, duration, and combustion conditions remain unknown. Other unrecognized environmental or airborne toxins present at the exposure site cannot be completely excluded as contributing factors. As with any single case report, a definitive causal relationship cannot be established; the temporal association reported here should therefore be regarded as hypothesis-generating rather than confirmatory and requires validation through dedicated toxicological and epidemiological investigation.

Although formal causality assessment tools, such as the World Health Organization-Uppsala Monitoring Centre (WHO-UMC) system or the Naranjo Adverse Drug Reaction Probability Scale, are commonly used to evaluate suspected adverse reactions, their application in this case is limited because they have not been validated for accidental environmental exposure to plant smoke [9,10]. Consequently, the observed association should be interpreted as hypothesis-generating rather than confirmatory.

## Conclusions

We described a case of new-onset convulsive status epilepticus occurring within minutes of accidental inhalation of smoke generated from burning Ageratum conyzoides. Despite extensive diagnostic evaluation, no alternative etiology was identified. Although causality cannot be established, this temporal association suggests a possible neurotoxic effect of inhalational exposure to Ageratum conyzoides smoke that warrants further investigation. Clinicians should consider environmental and plant-related toxic exposures when evaluating unexplained new-onset status epilepticus. Further toxicological and epidemiological studies are needed to better characterize the neurological effects of Ageratum conyzoides smoke exposure.
